# Uncovering potential diagnostic and pathophysiological roles of α‐synuclein and DJ‐1 in melanoma

**DOI:** 10.1002/cam4.6900

**Published:** 2024-01-08

**Authors:** Agathe Quesnel, Leya Danielle Martin, Chaimaa Tarzi, Vasileios P. Lenis, Nathan Coles, Meez Islam, Claudio Angione, Tiago F. Outeiro, Ahmad A. Khundakar, Panagiota S. Filippou

**Affiliations:** ^1^ School of Health & Life Sciences Teesside University Middlesbrough UK; ^2^ National Horizons Centre Teesside University Darlington UK; ^3^ School of Computing, Engineering & Digital Technologies Teesside University Middlesbrough UK; ^4^ Centre for Digital Innovation Teesside University Middlesbrough UK; ^5^ Translational and Clinical Research Institute, Faculty of Medical Sciences Newcastle University Newcastle upon Tyne UK; ^6^ Department of Experimental Neurodegeneration, Center for Biostructural Imaging of Neurodegeneration University Medical Center Göttingen Germany; ^7^ Max Planck Institute for Multidisciplinary Sciences Göttingen Germany; ^8^ Deutsches Zentrum für Neurodegenerative Erkrankungen (DZNE) Göttingen Germany

**Keywords:** chemotherapeutic drugs, diagnosis, DJ‐1, melanoma, molecular docking, α‐Synuclein

## Abstract

**Background:**

Melanoma, the most lethal skin cancer type, occurs more frequently in Parkinson's disease (PD), and PD is more frequent in melanoma patients, suggesting disease mechanisms overlap. α‐synuclein, a protein that accumulates in PD brain, and the oncogene DJ‐1, which is associated with PD autosomal recessive forms, are both elevated in melanoma cells. Whether this indicates melanoma progression or constitutes a protective response remains unclear. We hereby investigated the molecular mechanisms through which α‐synuclein and DJ‐1 interact, suggesting novel biomarkers and targets in melanoma.

**Methods:**

The Cancer Genome Atlas (TCGA) expression profiles derived from UCSC Xena were used to obtain α‐synuclein and DJ‐1 expression and correlated with survival in skin cutaneous melanoma (SKCM). Immunohistochemistry determined the expression in metastatic melanoma lymph nodes. Protein–protein interactions (PPIs) and molecular docking assessed protein binding and affinity with chemotherapeutic drugs. Further validation was performed using in vitro cellular models and ELISA immunoassays.

**Results:**

α‐synuclein and DJ‐1 were upregulated in primary and metastatic SKCM. Aggregated α‐synuclein was selectively detected in metastatic melanoma lymph nodes. α‐synuclein overexpression in SK‐MEL‐28 cells induced the expression of DJ‐1, supporting PPI and a positive correlation in melanoma patients. Molecular docking revealed a stable protein complex, with differential binding to chemotherapy drugs such as temozolomide, dacarbazine, and doxorubicin. Parallel reduction of both proteins in temozolomide‐treated SK‐MEL‐28 spheroids suggests drug binding may affect protein interaction and/or stability.

**Conclusion:**

α‐synuclein, together with DJ‐1, may play a role in melanoma progression and chemosensitivity, constituting novel targets for therapeutic intervention, and possible biomarkers for melanoma.

## 1 INTRODUCTION

Melanoma is the most lethal form of skin cancer with an enhanced ability to metastasize to distinct organs via hematogenous or lymphatic circulation.^1^ Skin cutaneous melanoma (SKCM) has a high degree of malignancy and invasiveness, causing over 72% of deaths. General models of SKCM progression begin in the melanocyte, progressing to in‐situ and finally the invasive melanoma.^1^ However, the exact mechanism of SKCM tumorigenesis and metastasis remains unclear.

Early detection is crucial, as the melanoma can grow and spread to other body areas, leading to advanced/metastatic melanoma which remains difficult to treat.^2^ While recently developed MAPK pathway inhibitors and immune checkpoint mediators represent meaningful progress in the treatment of advanced melanomas,^2^, ^3^ some patients still develop resistance and succumb to metastatic disease. Clinical studies using chemotherapeutic agents as monotherapy, or in combination, have not significantly improved response rates, with temozolomide (TMZ) as one of those chemotherapy drugs.^4^, ^5^ Consequently, there is an urgent need for novel therapeutic targets that improve chemosensitivity and avoid chemoresistance, as well as biomarkers for earlier diagnosis before the onset of advanced metastatic melanoma, which almost always proves fatal.

Neurodegenerative diseases and cancer are age‐associated disorders that are among the leading causes of cancer death worldwide.^6^ Parkinson's disease (PD), is pathologically defined by the selective degeneration of dopaminergic neurons in the substantia nigra pars compacta, and by the accumulation of proteinaceous inclusions known as Lewy bodies and Lewy neurites.^7^ Cancer, on the contrary, is caused by uncontrolled cell proliferation. Strikingly, melanoma occurs more frequently in PD patients, and PD is more frequent in melanoma patients.^8^ Many hypotheses have been drawn to explain the co‐occurrence of both diseases and shared genetic risk factors,^9^ but the underlying mechanisms are still unknown.^6^

Alpha‐synuclein (α‐syn), a major component of Lewy bodies and Lewy neurites in the brains of PD patients^10^ is elevated in malignant melanoma cells.^11^, ^12^ DJ‐1/*PARK7*, another PD‐associated protein, is an oncogene^13^ overexpressed in melanoma, but it is unclear whether this contributes to melanoma progression or, is perhaps part of a protective response.^14^, ^15^ In PD, DJ‐1 protects cells from oxidative stress and interacts with α‐syn, reducing its aggregation and toxicity, which may also take place in melanoma.^14^

α‐syn is a clinically important molecule since gene mutations and copy number variations have been linked to familial PD.^16^, ^17^ Biomarker‐based studies have detected and quantified α‐syn levels in PD (total, oligomeric/aggregated, or modified forms such as post‐translational modifications, PTMs).^10^, ^18^, ^19^, ^20^ PTMs, especially phosphorylation, have emerged as important determinants of the physiological and pathological functions of α‐syn. α‐syn has some experimentally proven phosphorylation sites^21^, ^22^, ^23^ with S129 being the best studied.^21^ The close association between specific PTMs and pathological aggregates could be used to detect, and monitor pathology in melanoma, like PD.^21^ Strikingly, recent studies suggest that some PTMs (i.e pS129‐α‐syn) seen in pathological aggregates may occur after α‐syn aggregation or inhibition of seeded fibril formation,^24^ therefore the exact mechanism needs to be further elucidated.

The effect of a drug in the expression of a protein target may result in resistance to conventional chemotherapy and/or targeted therapies.^25^, ^26^ Melanoma drug chemoresistance is one of the main features in consequent mortality.^27^ It is unclear whether altered α‐syn and/or DJ‐1 expression is a generalized feature of advanced melanomas and whether these genes along with or in combination have a functional contribution to melanoma progression and drug response. One common chemotherapeutic drug, doxorubicin, is known to interact with some physiological proteins and induce their destabilization.^28^ More importantly, this drug was found to interact with the central aggregation‐prone region of α‐syn and induce destabilization leading to its aggregation.^29^

In this study, we asked whether doxorubicin and other selected chemotherapeutic drugs used in melanoma (such as temozolomide) and its analog dacarbazine: (i) have the ability to stably bind α‐syn and DJ‐1 (alone or as complex) and (ii) whether this binding induces destabilization and further in vitro degradation. Thus, by combining bioinformatic with in vitro validation approaches, we aim to explore the molecular mechanisms of α‐syn and DJ‐1 in melanoma progression and to evaluate their diagnostic and prognostic potential and the possible impact of chemotherapy response in melanoma skin cancer.

## 2 MATERIALS AND METHODS

### 2.1 Clinical samples of patients and ethical approval

Formalin‐fixed paraffin‐embedded lymph node slides (3 metastatic malignant melanoma lymph nodes TNM8 Stage 3 and 3 non‐metastatic lymph nodes used as control (prostate neoplasm; pN0 lymph node status)) were obtained from NovoPath Biobank (Newcastle, UK). The melanoma stage for each case was pathologically determined, according to the established criteria.

NHS‐HRA‐North‐East‐Newcastle & North Tyneside 1 of NovoPath Biobank Newcastle Research Ethics Committee approved the sample collection (REC Reference 17/NE/0070) of the current study. Informed consent was collected for each patient and all procedures followed the Declaration of Helsinki.

### 2.2 Immunohistochemistry

Lymph node fixed sections were dewaxed by serial incubation in xylene (Sigma‐Aldrich) and decreasing concentrations of ethanol solution. Antigen retrieval was performed by boiling the slides in sodium citrate buffer. Endogenous peroxidase blockage was performed by 20 min incubation in 3% H_2_O_2_ at room temperature. Blocking was performed for 1 hour at room temperature in Tris‐buffered saline with 2%‐BSA/10%‐horse serum solution.

Primary antibodies diluted into the blocking solution were incubated overnight at 4°C, with the following dilutions: mouse monoclonal anti‐aggregated α‐synuclein (clone 5G4 MABN389; 1:1000 dilution, Millipore), rabbit monoclonal anti‐α‐synuclein phospho (Ser129) (clone Ab51253, 1:300 dilution, Abcam, Cambridge), rabbit monoclonal recombinant anti‐α‐synuclein aggregate antibody [MJFR‐14‐6‐4‐2]‐conformation‐specific, capturing filament and/or aggregated α‐syn (clone Ab209538, 1:2000 dilution, Abcam, Cambridge), and mouse monoclonal anti‐DJ‐1/*PARK7* (clone A16125E, 1:500 dilution, BioLegend). Universal probe, horseradish peroxidase, and diaminobenzidine tetrahydrochloride (Menarini Diagnostic kit, Winnersh, UK) were used for signal detection, as previously.^30^, ^31^ Images were taken with a Leica microscope DM75 (Leica microsystem, UK) at a magnification of 20×. Haematoxylin and eosin (H&E) staining was performed on a representative slide for each case. Slides were scored by the investigators, in a blind mode and the intensity and proportion of expressing cells were considered for analysis. Scoring was performed from 10 views at a magnification of 20× for each slide and each protein marker.

### 2.3 Gene expression and survival analysis using publicly available data

Gene expression patterns of *SNCA* and DJ‐1/*PARK7* were explored in a pan‐cancer analysis, using the GEPIA webserver^32^, ^33^, ^34^ based on tumor and normal samples from the TCGA and GTEx databases (accessed 21 April 2023). RNA‐seq data represented as transcripts per million (TPM) indicate the gene expression profile across all tumor samples and paired normal tissues.

The differences in *SNCA* and DJ‐1/*PARK7* gene expression between normal, tumor, and metastatic tissues in SKCM were investigated using transcriptomic TCGA data accessed via the Xena UCSC portal (http://xena.ucsc.edu).^35^ Sequencing reads obtained from the Cancer Genomics Project^36^ using the illumina® platform, were normalized by RSEM^37^ and logged transformed (log2). Statistical analysis was conducted with the Wilcoxon non‐parametric rank sum test between groups (normal vs tumor; normal vs primary tumor; normal vs metastatic; primary tumor vs metastatic) using the R version 3.5.1. Results were visualized as boxplots.

*SNCA* and DJ‐1/*PARK7* gene expression data from human cancer cell lines were downloaded from Cancer Cell Line Encyclopedia (CCLE) (accessed on 21 April 2023) and R (v3.5.1) was used to visualize the results in graphs.

GEPIA2^33^ was also employed to explore survival curves for overall survival (OS) for each protein in SKCM based on the Kaplan–Meier plotter data resource. A survival map across TCGA tumors was also generated for OS. A cut‐off value median of 50% was set as the expression threshold for separating high‐ and low‐expression clinical cohorts and log‐rank test was used (log‐rank *p* < 0.05).

### 2.4 Cell lines and culture conditions

A375 (CRL‐1619) and SK‐MEL‐28 (HTB‐72™) human malignant melanoma cell lines were received from the American Type Culture Collection (ATCC), certified by short‐tandem repeat DNA profiling authentication and a negative test for mycoplasma contamination.

A375 and SK‐MEL‐28 cell lines were cultured in ATCC‐formulated Dulbecco's Modified Eagle's Medium (A375) and ATCC‐formulated Eagle's Minimum Essential Medium (SK‐MEL‐28) supplemented with 10% fetal bovine serum and 1× antibiotic‐antimycotic (Gibco, Fisher Scientific). Melanoma cell lines were cultured in a humidified incubator with 5% CO_2_ at 37°C.

### 2.5 Transient α‐syn overexpression in SK‐MEL‐28 and A375 melanoma cells

SK‐MEL‐28 and A375 melanoma cells were transiently transfected with the FuGENE HD transfection reagent (Promega). 1 × 10^5^ cells were seeded before transfection into 12‐well plates. Cells were cultured in Opti‐MEM‐reduced serum medium supplemented with 5% FBS and 1× antibiotic‐antimycotic. pcDNA3.1+−wild‐type‐α‐synuclein plasmid (kindly provided by Prof Outeiro's lab) was used for transfection of the melanoma cells at a ratio of plasmid (μg): transfection reagent (μL) of 1:6 in Opti‐MEM medium. Mock transfection was used as a control. After 24 h transient transfection, the cell's supernatant (secretome) was collected and protein expression of α‐syn and DJ‐1 was measured by ELISA immunoassays.

### 2.6 ELISA immunoassays

α‐syn and DJ‐1/*PARK7* concentrations in melanoma cell supernatants and extracts were measured using the human α‐syn SimpleStep ELISA® Kit (ab260052, Abcam, Cambridge) and the human PARK7 SimpleStep ELISA® Kit (ab215535, Abcam, Cambridge) according to the manufacturer's instructions. Samples were diluted 1:2 prior to ELISA and α‐syn and DJ‐1 protein concentration (ng/mL) was estimated from each ELISA using a standard curve.

### 2.7 SK‐MEL‐28 melanoma spheroid formation and treatments

SK‐MEL‐28 multicellular spheroids were generated using the “hanging drop” method.^38^, ^39^ Briefly, cells were cultured and added in suspension at 2–2.5 × 10^4^ cells/mL. Next, around 500 cells were placed on the inside cover of a 100‐mm culture dish as hanging drops (20 μL) and left for 48 h. The formed spheroids were transferred into a 96‐well plate,^38^, ^39^ and culture medium was then added (in the absence (DMSO used as vehicle; control) or presence of TMZ (Sigma, #T2577) at 80 μg/mL (TMZ‐C1) or 200 μg/mL [TMZ‐C2]). Images were taken after 24 h and every other day, using a Leica inverted DMi1 microscope. The spheroid surface area was measured (12 spheroid measurements/condition) using the ICY Bioimage analysis software (https://icy.bioimageanalysis.org/). Ten spheroids (day 6) were selected per each condition for further ELISA measurements.

### 2.8 Molecular docking

The structures of α‐syn and DJ‐1 were obtained from AlphaFold prediction (https://alphafold.ebi.ac.uk/). Docking studies of the binding modes between: (i) α‐syn and DJ‐1, (ii) each protein separately with each chemotherapeutic drug, or (iii) the complex with each drug, were conducted. Computational protein‐ligand docking to predict the bound conformations and free binding energy for small‐molecule ligands to macromolecular targets, as well as protein–protein docking, was used with the AutoGrid 4.0 and AutoDock 4.0 software.^40^ According to the docking score, the best predicted binding mode was selected to analyze the detailed interaction network between the two proteins or protein(s)/drug. The coordinated files and corresponding information were created in PDBQT format using AutoDockTools (version 1.5.7).^41^ Subsequently, the ligands were prepared for docking runs through PyMOL.^42^ For each binding site, every ligand atom was analyzed for its interaction energy with the receptor, which was discretized using a grid map. Each indicated docking pose including docking score, RMSD, estimated inhibition constant, and other parameters enabled the direct analysis of configuration/score relationships. The lower binding affinity energy estimation of the receptor and the ligand (best predicted binding mode) was visualized, analyzed, and mapped using the PyMOL molecular visualization system.^42^

### 2.9 Protein–protein interaction networks and correlation analysis

The STRING database^43^ was accessed on 21 April 2023 to construct the *SNCA (encoding* α‐syn)‐mediated protein–protein interactions (PPIs) network with the following parameters: “*SNCA*” and organism (“Homo sapiens”), minimum required interaction score (highest confidence 0.90), max number of interactors (“no more than 10 interactors” in first shell) and all active interaction sources. GeneMANIA^44^ was also used (accessed on 21 April 2023) to create an interactive functional association network for *SNCA*. Finally, Venny 2.1^45^ (https://bioinfogp.cnb.csic.es/tools/venny/) was employed to conduct an intersection analysis to compare GeneMANIA and STRING with the generation of Venn diagrams. The Spearman correlation between the expression of the two genes (*SNCA* and DJ‐1/*PARK7*) on the SKCM primary tumor (*N* = 102) and the metastatic tumor (*N* = 367) was also explored in the TCGA data using the R version 3.5.1 (Spearman's *p*: positive correlation; *p* < 0.01, ρ > 0).

### 2.10 Statistical analysis

GraphPad Prism 9 (V9.4.1, GraphPad Software, CA, USA) and the R version 3.5.1 statistical packages were used for the statistical analysis of the current study. For ELISA and spheroid surface measurements, one‐way ordinary ANOVA followed by Tukey's multiple comparisons was used. All p‐values given were 2‐sided and a *p*‐value ≤0.05 at a 95% confidence interval was considered statistically significant.

## 3 RESULTS

### 3.1 Expression patterns and survival prognosis of *SNCA* and *PARK7* in SKCM

**FIGURE 1:**
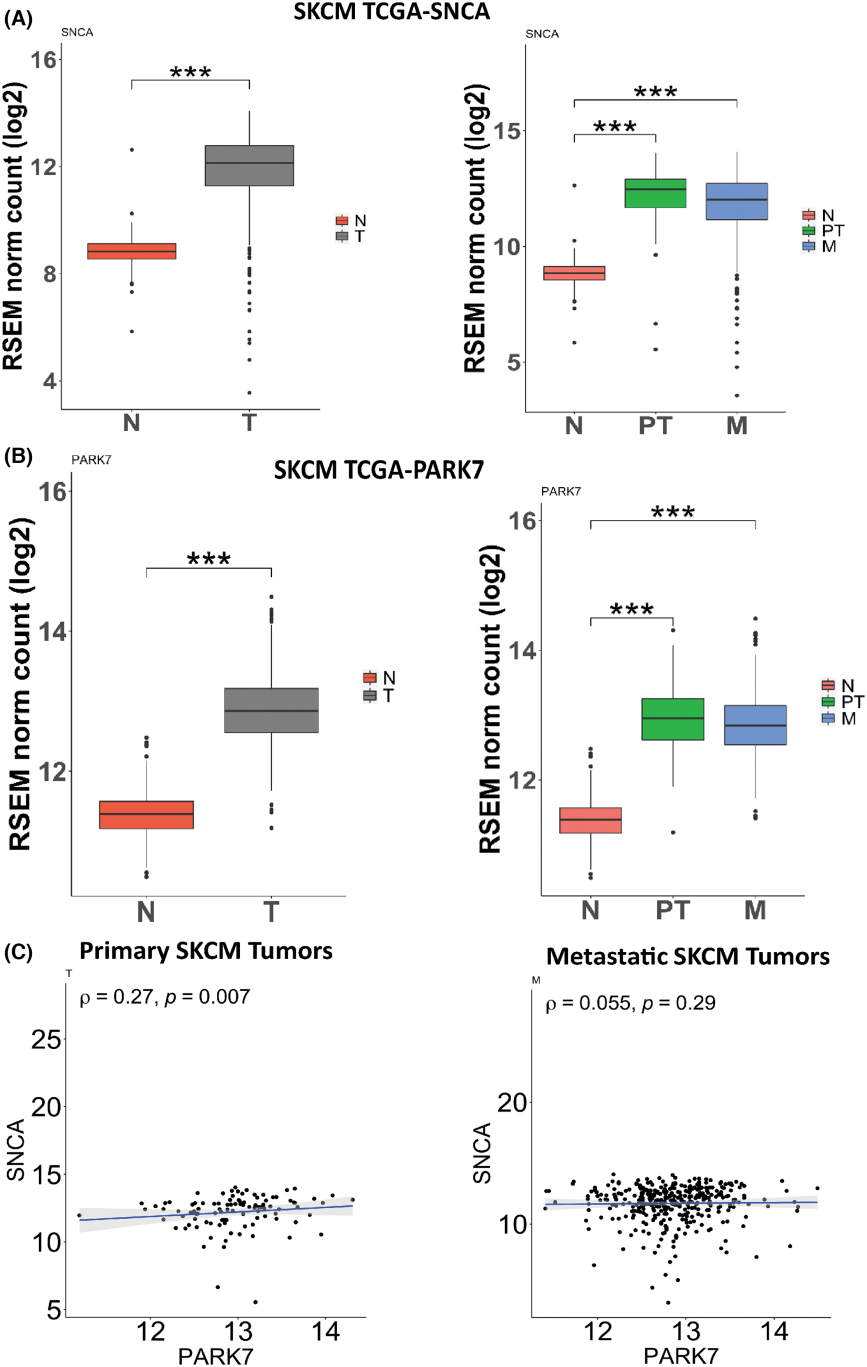
Expression profiles of *SNCA* and *PARK7* in skin cutaneous melanoma (SKCM). (A, B) RNA sequencing transcriptomic TCGA data from Xena UCSC portal were used to compare Normal (N) (*n* = 556) with Tumor (T; primary and metastatic) (*n* = 469) (A), and Normal (N) (*n* = 556) with Primary Tumor (PT) (*n* = 102) or Metastatic (M) (*n* = 367) (B). Both *SNCA* and *PARK7* expression levels are significantly upregulated in SKCM tumor samples (primary and metastatic) compared to normal skin tissues. Wilcoxon rank sum non‐parametric test was applied between groups for statistical significance (***p < 0.001). (C) Positive Spearman correlation was observed between *SNCA* and *PARK7* gene expression in SKCM‐primary (*N* = 102) (Spearman's *ρ*: positive correlation (*ρ* > 0, *p* < 0.01) whereas not in SKCM‐metastatic tumors.

Firstly, we explored the gene expression profiles of *SNCA* (encoding for α‐syn) and *PARK7* (encoding for DJ‐1) in different cancer types using TCGA datasets (Figure S1). Strikingly, the expression of α‐syn was significantly higher only in two cancers, SKCM and pancreatic adenocarcinoma (PAAD), whereas it was reduced in 15 cancers. Compared to normal tissues, the expression of *SNCA* was significantly upregulated in SKCM and very notable compared with that in other types of cancer. *PARK7*, in contrast, was upregulated in four cancer types, including SKCM when compared to normal tissues, and was downregulated only in acute myeloid leukemia (LAML; Figure S1B). Since both *SNCA* and *PARK7* appeared to be significantly up‐regulated in SKCM in the pan‐cancer analysis, we further validated these results using the TCGA datasets (Figure 1).

Compared to normal skin tissue (*N* = 556), the overall tumors (*T* = 469), primary tumors (PT = 102), as well as metastatic (M = 367) of SKCM patients, demonstrated significantly higher *SNCA* and *PARK7* expression (Figure 1A,B). No statistical significance was observed between primary tumors (PT) and metastatic (M) (Figure 1).

Next, we investigated whether *SNCA* and *PARK7* expression are related to the survival prognosis in patients from various cancer types including SKCM (Figure S2). Focusing on the OS map, high *SNCA* expression was linked to poor prognosis for only three cancer types: head and neck squamous cell carcinoma (HNSC), stomach adenocarcinoma (STAD), and SKCM. Poor prognosis in SKCM based on OS was significantly correlated with high α‐syn expression (LogRank *p* = 0.03; Figure S2B). Conversely, *PARK7* expression did not appear to have a significant correlation to the survival prognosis of SKCM patients. Likewise, no significant correlation was observed between high *PARK7* expression and SKCM percent survival for SKCM (LogRank *p* = 0.31; Figure S2A).

We further explored the correlation observed between *SNCA* and *PARK7* expression in SKCM primary tumor patients using the transcriptomic TCGA data (*N* = 102). Spearman's positive correlation was observed in SKCM primary (Spearman's *p*: positive correlation *p* < 0.01, *ρ* = 0.27; Figure 1C, left panel) but not in metastatic SKCM tumors (*N* = 367) of melanoma patients (Figure 1C, right panel; *p* = 0.29, *ρ* = 0.055).

Upon further analysis of an independent RNA sequencing dataset (GSE112509) of 80 samples (primary melanomas [*n* = 57] and benign melanocytic nevi [*n* = 23]),^46^ no statistical significance was observed for *SNCA* expression between primary melanoma and melanocytic nevi samples (Wilcoxon Rank Sum test, *p* = 0.1424). For *PARK7*, a significantly higher expression was observed in primary melanomas (*p* = 0.00272; Figure S3). In addition, a good positive correlation (spearman's test) between *SNCA* and *PARK7* expression was observed in primary melanomas (*ρ* = 0.48, *p* = 0.00019), but not in benign melanocytic nevi (*ρ* = 0.34, *p* = 0.12).

### 3.2 α‐syn and DJ‐1 protein expression in metastatic melanoma lymph nodes

**FIGURE 2:**
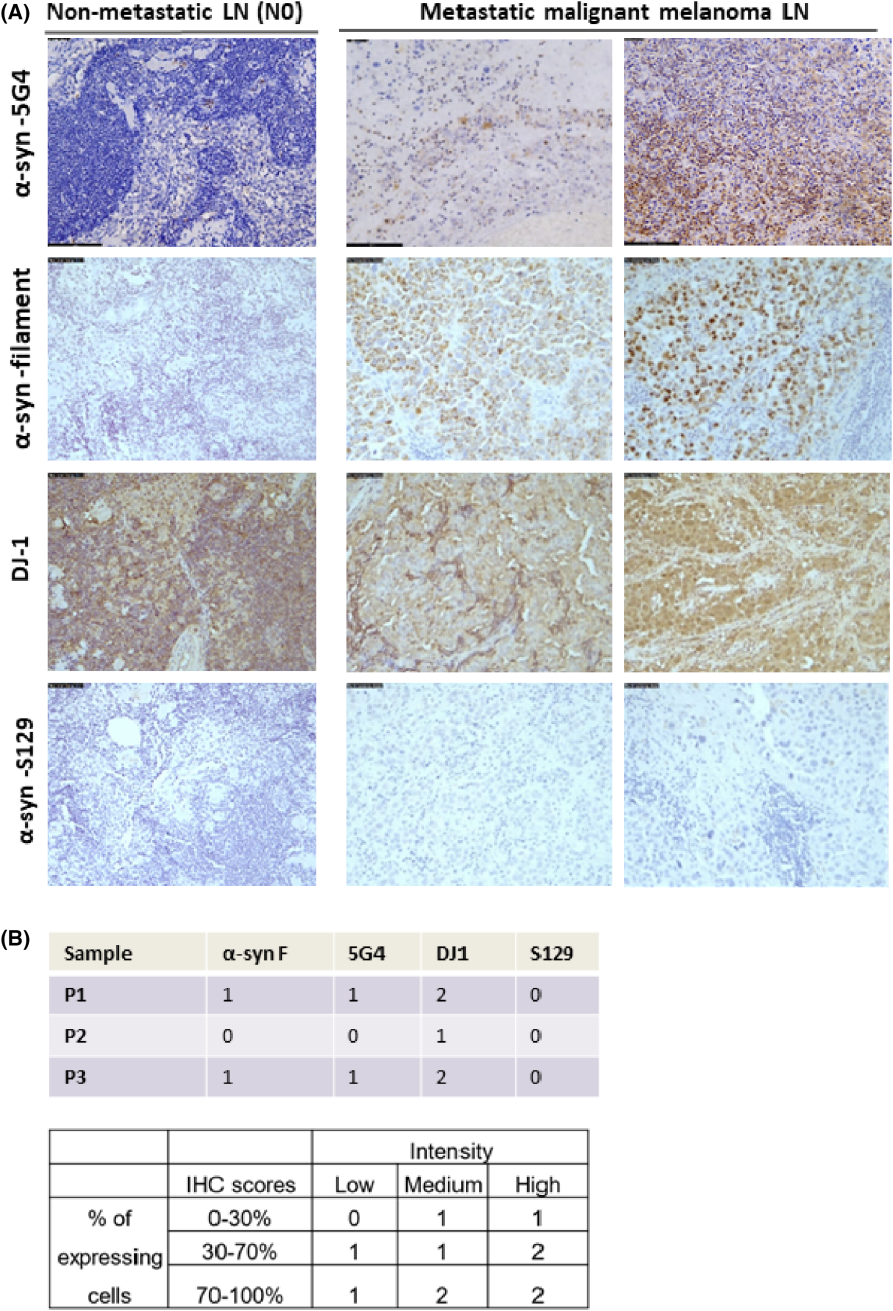
α‐syn and DJ‐1 protein expression in lymph nodes from metastatic melanoma patients. (A) Immunohistochemistry was performed in lymph nodes of patients with metastatic malignant melanoma with antibodies capturing α‐syn aggregated forms (α‐syn filament and α‐syn‐5G4), the α‐syn‐Ser129 phosphorylated form (α‐syn‐S129), and DJ‐1. Representative images of stained lymph node tissue sections from 2 metastatic malignant melanoma patients (right panel) and a patient with non‐metastatic (N0) carcinoma lymph nodes (control, left panel) are shown. Magnification, 20×. (B) The slides were categorized into three scores according to the percentage of area and intensity of staining. Score 2 represents the highest expression. α‐syn aggregated forms were highly expressed in metastatic malignant melanoma compared to non‐metastatic lymph nodes. Expression of α‐syn phosphorylation form was almost absent in metastatic melanoma lymph nodes, whereas DJ‐1 was expressed in both control and metastatic melanoma lymph nodes.

Next, we performed immunohistochemistry analysis in fixed tissue biopsies from patients with metastatic malignant melanoma (TNM8 Stage 3) and non‐metastatic lymph nodes (prostate neoplasm; pN0 lymph nodes) (control) to further investigate: (i) the differential expression and distribution of α‐syn and DJ‐1 in metastatic melanoma lymph nodes compared to non‐metastatic non‐melanoma lymph nodes and (ii) whether specific pathological forms of α‐syn may be detected in melanoma lymph node metastasis. Antibodies that detect PD‐related pathological and aggregated/filamentous forms of α‐syn were used for staining and the tumors were categorized using three IHC scores (0, 1, and 2) for each protein, depending on both the intensity and percentage of expressing cells (Figure 2), as previously described.^31^ α‐syn aggregated forms were detected at higher levels in metastatic melanoma lymph nodes compared to control lymph nodes (with low to no detection of α‐syn) after staining with α‐syn‐5G4, an antibody that recognizes aggregated/filamentous α‐syn (Figure 2A,B). On the contrary, DJ‐1 was not specifically expressed in metastatic melanoma lymph nodes, as it was also detected in the control lymph nodes (Figure 2A) in agreement with Human Protein Atlas (https://www.proteinatlas.org/) lymph node expression.

We also assessed the presence of phosphorylated α‐syn on serine‐129 (α‐syn‐S219), as this is considered a pathological form of α‐syn in PD. Interestingly, phosphorylated S129 α‐syn was almost absent in metastatic malignant melanoma lymph nodes, suggesting that this phosphorylation form may not be directly implicated in α‐syn aggregation in melanoma metastasis (Figure 2A,B).

### 3.3 Expression of α‐syn and DJ‐1 in melanoma cell lines

Given that α‐syn and DJ‐1 are both significantly upregulated in SKCM and may participate in a common mechanism of melanoma progression, we investigated the expression of the genes encoding for these proteins in a variety of melanoma cell lines, with further in vitro validation of selected cell lines at the protein level.

**FIGURE 3:**
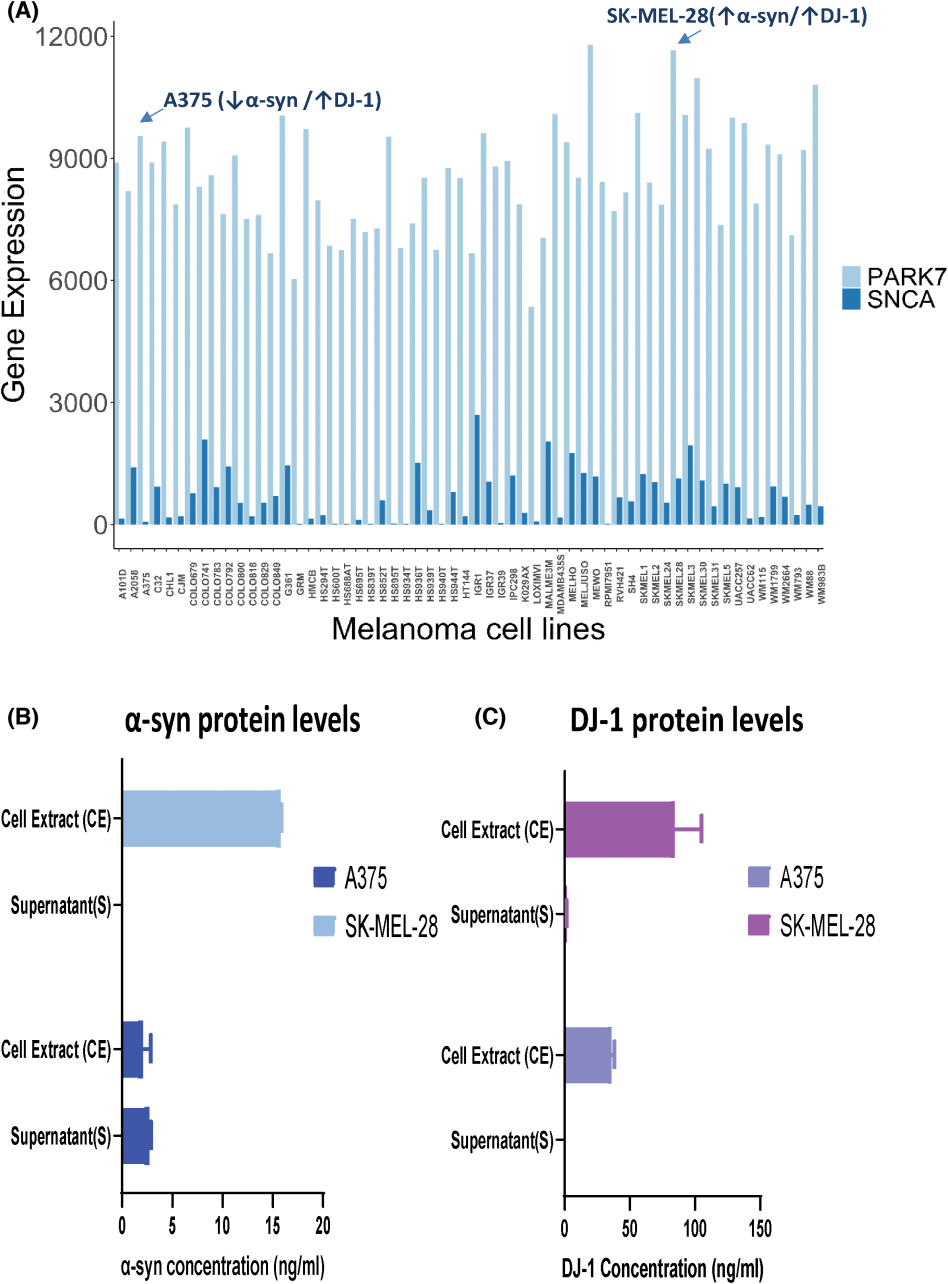
Expression profile of α‐syn and DJ‐1 in melanoma cell lines. (A) Gene expression comparative bar plot for *SNCA* (encoding α‐syn) and *PARK7* (encoding DJ‐1) using the Cancer Cell Line Encyclopedia datasets via the Betastasis platform. (B, C) Validation of selected melanoma cell lines (A375 and SK‐MEL‐28) at the protein level using ELISA immunoassays. α‐syn (B) and DJ‐1 (C) protein levels were measured as ng/mL in the supernatants (secretome, S) and cell extracts (CE) of the selected melanoma cell lines. Mean values with standard deviation (SD) are indicated in the bar graphs.

According to the gene expression data from cell lines downloaded from the Cancer Cell Line Encyclopedia repository (Betastasis), *SNCA* is differentially expressed in various melanoma cell lines with very low to high expression levels, depending on cell line type (Figure 3A). *DJ‐1*, on the contrary, appears with no significant variations at the gene expression level among the various melanoma cell lines (Figure 3A).

Two melanoma cell lines (SK‐MEL‐28 and A375), that differentially express the two proteins, were chosen for further validation at the protein level using ELISA immunoassays. *SNCA* is expressed at moderate‐high gene expression level in SK‐MEL‐28 (Gene expression intensity = 1135), whereas expression appears at very low levels in A375 (Gene expression intensity = 64.98; expression intensities arbitrarily defined as <1000: low‐moderate; 1000–2000: moderate‐high; >2000: high expression). *DJ‐1*, on the contrary, appears with relatively high expression levels for both SK‐MEL‐28 (Gene expression intensity =11,657) and A375 (Gene expression intensity = 9548; Gene expression intensities >9500 were arbitrarily defined as high expression) (Figure 3A). Consistently with the mRNA levels (Figure 3A), α‐syn intracellular protein levels were higher in SK‐MEL‐28 compared to A375 (Figure 3B), and intracellular DJ‐1 levels (Figure 3C).

Interestingly, α‐syn and DJ‐1 appeared exclusively intracellularly in SK‐MEL‐28 (in the cell extract), whereas no secreted proteins (supernatant) were detectable, under the experimental conditions (Figure 3B,C). Contrarily, the lower levels of α‐syn in the A375 compared to SK‐MEL‐28, appeared with an equal distribution for both α‐syn intracellular and secreted forms (Figure 3B). DJ‐1, on the contrary, was expressed exclusively as intracellular in the A375 melanoma cell line (Figure 3C).

### 3.4 α‐syn PPIs and association with DJ‐1

Previous studies have suggested the interaction of α‐syn with DJ‐1 in PD^14^, ^47^ but their connection in melanoma has not been studied. To address this, we conducted a three‐pronged analysis by: (i) identifying the association of α‐syn (*SNCA*) and DJ‐1(*PARK7*) through the α‐syn PPI networks, (ii) further investigating their interaction by identifying the protein‐binding domains via molecular docking studies, and (iii) examining their co‐occurrence in melanoma cells.

**FIGURE 4:**
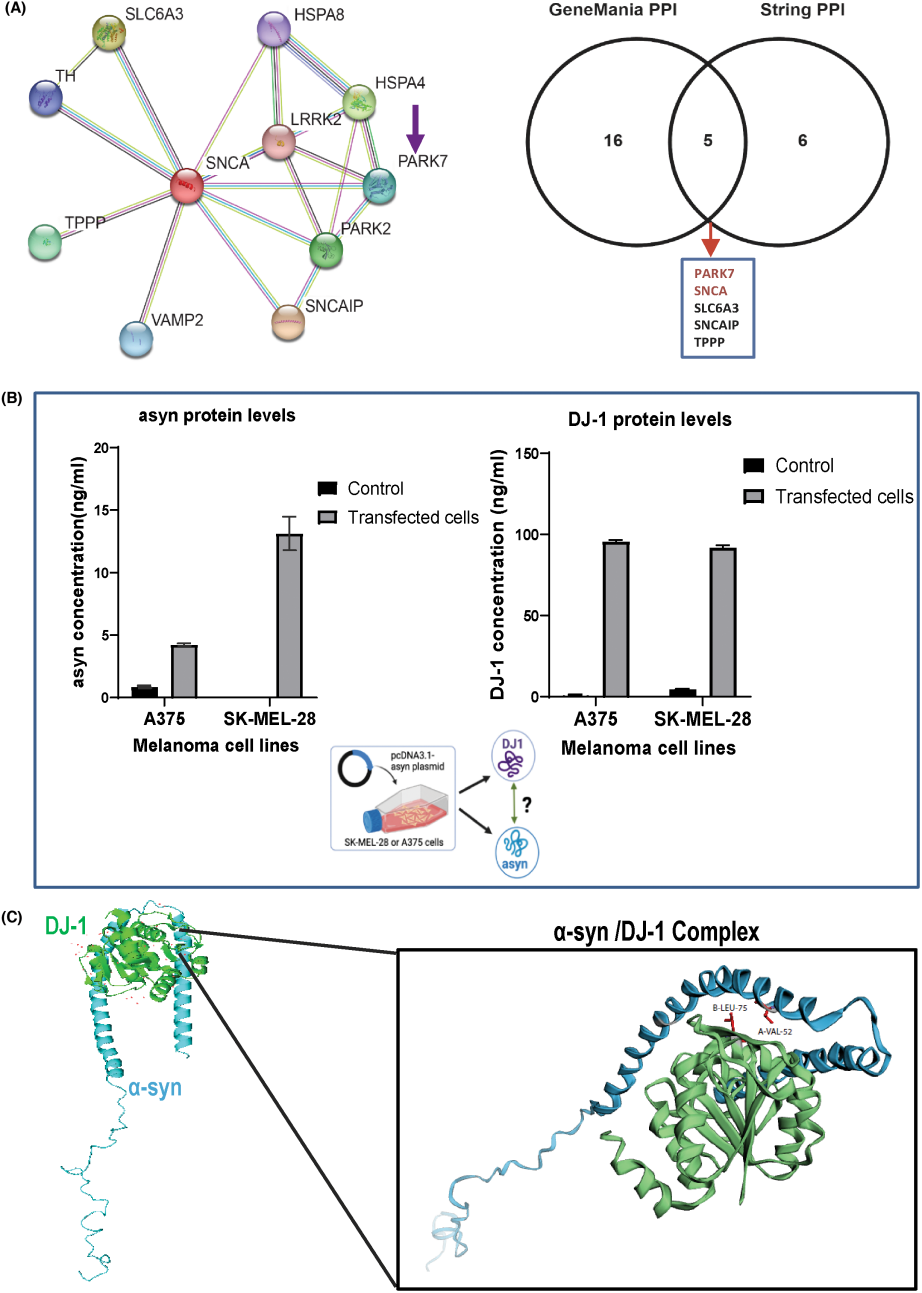
Protein–protein interactions and association of the PD‐related α‐syn with DJ‐1. (A) Interaction and co‐occurrence protein partners of α‐syn (*SNCA*) were identified with String and GeneMANIA databases. Circles displayed are indicated by nodes. Predicted functional partners are shown after considering co‐expression, co‐localization, genetic interactions, pathways, and physical interactions. Venn diagram employed to identify the commonly interactive protein partners of α‐syn, including DJ‐1(*PARK7*), based on GeneMANIA and STRING databases. (B) SK‐MEL‐28 and A375 melanoma cells were transfected with pcDNA3.1 + −wild‐type‐α‐synuclein plasmid and protein levels of α‐syn and DJ‐1 (ng/mL) were assessed in the cell supernatants (secretome) after 24 h of transfection. Mean values with standard deviation (SD) are indicated in the bar graphs. (C) Illustration of α‐syn (receptor, in blue) and DJ‐1 (ligand, in green) interacting protein domains and binding sites using a molecular docking approach. Interacting domain (zoom) that includes the two amino acid residues with the most stable binding affinity (LEU‐75 in the ligand‐DJ‐1 with binding free energy −2.55 kcal/mol and VAL‐52 in the receptor‐α‐syn with −3.74 kcal/mol).

To identify the potential binding partners of α‐syn, the PPI network was constructed using the GeneMANIA and STRING databases (Figure 4A). DJ‐1 was identified as an interacting partner for α‐syn through the results from STRING (10 identified proteins) and GENEMANIA (20 identified proteins). We next conducted an intersection analysis to compare the α‐syn PPI and identify the common α‐syn interacting partners based on both databases using a Venn diagram. Indeed, DJ‐1 was among the most potent interacting partners for α‐syn (Figure 4A).

Further transient transfection of both SK‐MEL‐28 and A375 cell lines led to the expected α‐syn overexpression and further caused a parallel increase of DJ‐1 protein levels, indicating the co‐occurrence of both proteins in melanoma cells (Figure 4B) in agreement with the positive correlation observed in SKCM melanoma patients (Figure 1C).

### 3.5 Molecular docking reveals the interaction binding of α‐syn with DJ‐1

**FIGURE 5:**
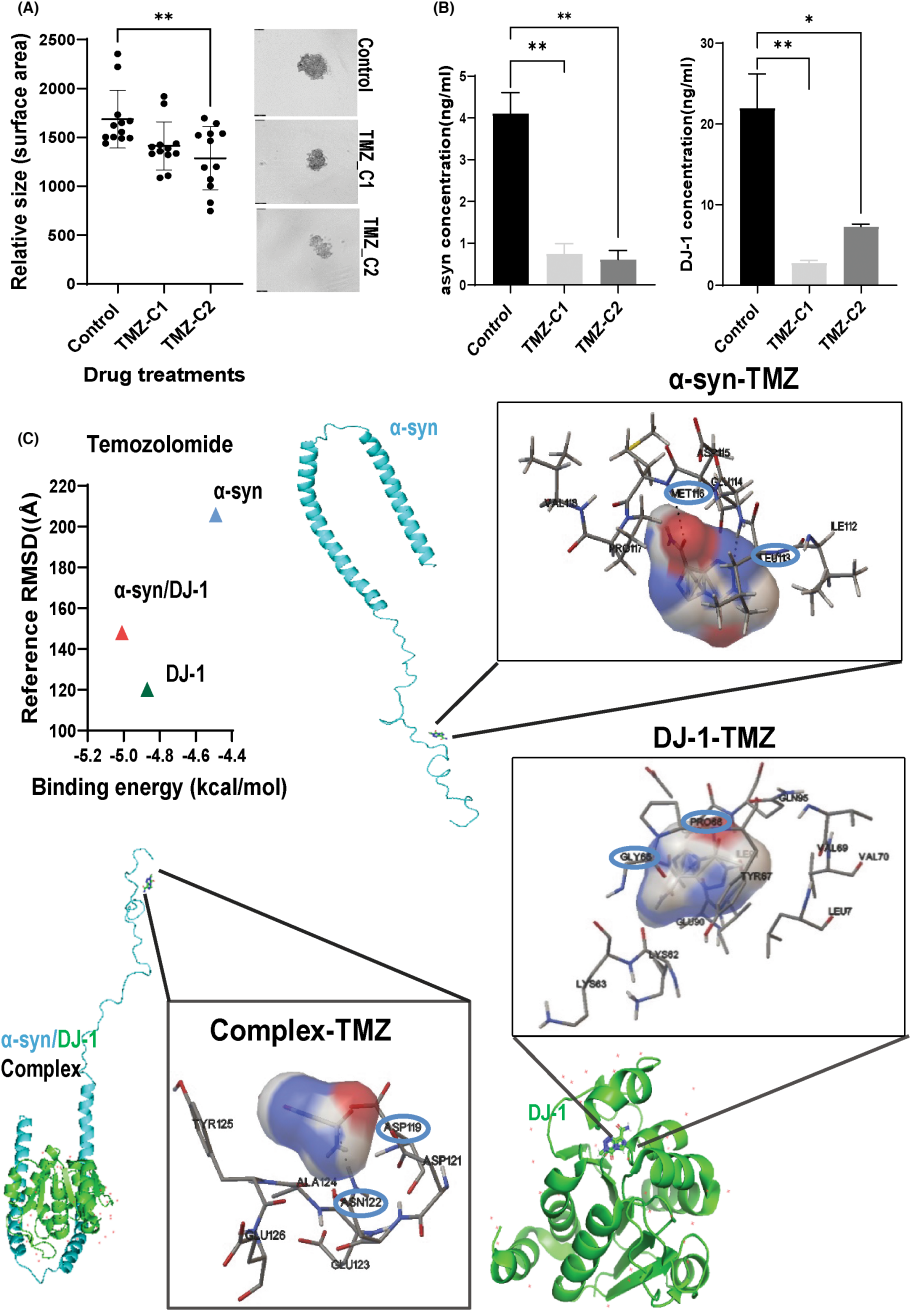
Effect of the chemotherapeutic drug temozolomide on α‐syn and DJ‐1 protein levels and interactions. (A) Representative images of the SK‐MEL‐28 spheroid generation in the absence (control) or presence of different temozolomide (TMZ) concentrations (TMZ‐C1: 80 μg/mL and TMZ‐C2: 200 μg/mL respectively). TMZ effect on the SK‐MEL‐28 spheroid model was explored by relative size spheroid measurements of the surface area (*N* = 12) and comparison between treated and untreated spheroids. Spheroid mean values (relative size surface area) with SD are indicated. (B) DJ‐1 and α‐syn protein levels (ng/mL) were measured in cell extract supernatants of the SK‐MEL‐28 spheroids grown in the absence (control) or presence of TMZ at 80 μg/mL (TMZ‐C1) or 200 μg/mL (TMZ‐C2) (*N* = 10 spheroids/condition). One‐way ordinary ANOVA followed by Tukey's multiple comparison test was used to test whether the mean concentration of proteins (B) or the relative size of spheroids (A) between control and treated were significantly different. Mean values ± SD are indicated in the graph. (C) Illustration of TMZ targeting α‐syn, DJ‐1 or their complex and their ligand‐targeted amino acids involved. TMZ molecule docked to the homology model of both proteins and their complex. The interacting amino acid residues for TMZ binding are: α‐syn‐TMZ (MET 116, LEU113), DJ‐1‐TMZ (PRO 66, GLY 65), and the complex (ASP 119, ASN 122). Hydrogen bonds are presented using dashed‐ lines and interacting amino acids with circled‐indicated points. Molecular docking for the binding energy (kcal/mol) of the ligand with the receptor (protein) and Reference RMSD (Å) were also estimated (Table S5) and plotted in terms of comparisons of the binding efficiencies.

To further verify the dynamic interactions and to gain insight into the binding domains by which those proteins interact, molecular docking studies were performed (Figure 4C; Tables S1 and S2). A docking protocol: rigid‐body docking was applied (PatchDock),^48^ followed by fast interaction refinement and scoring (FireDock).^49^ The server output (Table S1) shows all input solutions with each single input complex per row and global energy values. Refined complex structures were generated for up to 100 lowest energy candidates. Different complexes could be viewed simultaneously for comparisons with 3D PDB structures. The table was sorted by different energy terms, such as, among others, the attractive and repulsive van der Walls forces, the atomic contact energy (ACE), and the global binding energy (Table S1). The global binding energy was chosen as the main parameter to indicate the most stable protein complex interaction. α‐syn and DJ‐1 interact with a binding global energy of −17.13 kcal/mol which demonstrates a significant and stable complex PPI. The cartoon image shows the most stable with higher binding affinity complex of α‐syn‐DJ‐1 protein interaction (Figure 4C; the one with higher negative global energy, Table S1). This most stable complex was selected as the ligand‐receptor interaction to be part of the structure‐based drug design process later (Figure 5).

To further interpret the protein binding structures,^50^ the MM/GBSA method was employed (Table S2). The binding structures were analyzed and predicted the binding free energy and decomposed the free energy contributions to the binding free energy of a protein–protein complex in per‐residue (Table S2). Our results indicate that α‐syn (receptor) interacts via VAL‐52 (−3.74 kcal/mol total) with DJ‐1 (ligand) via LEU‐75 (−2.55 kcal/mol total Table S2) forming a stable complex (global binding energy −17.13 kcal/mol; Table S1; Figure 4C).

More importantly the broader region for α‐syn (VAL‐48 to‐VAL‐52; binding free energy: −2.75 to −3.74 kcal/mol respectively) that interacts with DJ‐1 according to our complex residue domain results (Figure 4C, Table S2) appears to be one of the critical suggested regions for α‐syn aggregation (N‐terminal residues from 36 to 42 and 45 to 57), according to other studies.^29^ In addition, the α‐syn region of interaction could also be considered as a highly hydrophobic region (VAL‐48, VAL‐49, HIE‐50, ‐GLY‐51, VAL‐52) (Table S2, Figure 4C).

### 3.6 Impact of chemotherapeutic drugs on α‐syn and/or DJ‐1

We first explored the drug sensitivity of TMZ in cancer cell lines in silico (Table S3), focusing on melanoma cell lines (Table S4) using the Genomics of Drug Sensitivity in Cancer (GDSC) project web portal^51^ (https://www.cancerrxgene.org/; accessed 2 January 2023). Among the 965 cancer cell lines screened and based on IC50 values comparison, SK‐MEL‐28 was among the cell lines with higher drug sensitivity and the first highly sensitive SKCM cell line (TMZ IC50:10.19 μM; GDSC2 dataset; Tables S3 and S4). Therefore, we chose the SK‐MEL‐28 cell line to further explore in vitro the impact of TMZ on α‐syn or DJ‐1 protein levels, which may imply a potential impact of those proteins in TMZ‐mediated chemosensitivity. A SK‐MEL‐28 malignant melanoma spheroid model expected to express at high levels of both proteins (Figure 3) was generated, and the effect of TMZ was examined on the growth and protein levels of α‐syn and DJ‐1. Spheroid size with surface area measurements (Figure 5A), indicated a reduction of the spheroid size upon TMZ treatment with each drug concentration (80 and 200 μg/mL respectively). Cell surface areas for each spheroid condition (*N* = 12 spheroids measurements/condition) showed reduction in treated compared to untreated spheroids (SK‐MEL‐28; 16% and 24% spheroid size reduction for 80 and 200 μg/mL respectively; Figure 5A). Parallel measurements of α‐syn and DJ‐1 protein levels in treated versus untreated spheroids (*N* = 10) indicated a significant reduction of both intracellular proteins in the SK‐MEL‐28 cells (Figure 5B).

One of the causes of the simultaneous reduction of both proteins could be the possible binding of TMZ in each protein separately or their complex that may affect the protein stability leading to possible degradation. Therefore, we conducted molecular docking studies to explore the binding of TMZ to those proteins (Figure 5C, Table S5). Docking experiments were performed with AutoDock4, and each docking pose included extra information such as the docking score, RMSD, and the estimated inhibition constant. Docked ligands and their corresponding binding poses were then ranked according to docking scores and the results of multiple docking runs are summarized (Table S5).

The binding of TMZ to alpha‐synuclein, DJ‐1, and their complex was performed through specific amino acid residues and drug interactions (Figure 5C). Our results confirmed that TMZ binds to each protein separately forming a stable drug‐protein complex (binding energy; α‐syn: −4.49 kcal/mol and DJ‐1: −4.87 kcal/mol) and for the complex with higher binding affinity through an α‐syn protein‐drug interaction (binding energy; −5.01 kcal/mol; Figure 5C, Table S5).

Next, we examined whether other chemotherapy drugs that also have been used for melanoma and/or other cancer treatments such as an analog of temozolomide, the dacarbazine, and a more general chemotherapeutic drug, doxorubicin that was shown to affect α‐syn aggregation,^29^ bind similarly to those proteins. Both drugs dacarbazine and doxorubicin, are bound efficiently with α‐syn, DJ‐1, and the complex (Figure S4, Table S5). Comparing the binding affinity of the three tested drugs according to the binding affinity energies, DJ‐1‐doxorubicin (−6.19 kcal/mol), followed by the complex‐doxorubicin (−5.59 kcal/mol) and the α‐syn‐doxorubicin (−5.17 kcal/mol) constitute the more stable complexes compared to the other two drugs. Interestingly, temozolomide has a stronger binding affinity compared to its analog dacarbazine (Table S5) with the complex‐temozolomide (−5.01 kcal/mol) showing a stronger binding affinity, followed by DJ‐1‐temozolomide (−4.87 kcal/mol) and α‐syn‐temozolomide (−4.49 kcal/mol).

## 4 DISCUSSION

Early‐stage melanoma is usually curable, but advanced malignant metastatic melanoma is almost always fatal with poor survival of patients.^52^, ^53^ Moreover, advanced melanoma patients may not respond or develop resistance to chemotherapy and/or immunotherapy,^53^ constituting current treatments insufficient. Therefore, there is an urgent need to detect the disease earlier and improve the efficiency of already used chemotherapeutic drugs, such as temozolomide and dacarbazine^4^, ^54^ in advanced metastatic malignant melanoma when assessing novel biomarkers and therapeutic targets. α‐syn and its aggregated forms, which constitute a pathological hallmark of PD,^55^, ^56^, ^57^ as well as the PD‐related protein DJ‐1, which is also an oncogene,^15^, ^58^, ^59^ may be involved in pathobiological mechanisms in melanoma onset and progression, like in PD.^6^, ^60^ Here, we aimed to explore the diagnostic and prognostic potential of both proteins and their possible implication in melanoma progression and treatments, using an in silico bioinformatic approach with further validation in *in vitro* cellular models and immunostaining/immunoassay approaches.

Our bioinformatic studies suggest that both α‐syn and DJ‐1 are upregulated in SKCM tumors (primary and metastatic), with high α‐syn expression correlated with worse clinical outcomes for patients. More importantly, human SKCM tumors exhibit the highest gene expression of α‐syn among other human cancers, suggesting a disease‐specific involvement and possible usefulness alone or in combination with DJ‐1 as potential biomarkers for melanoma diagnosis, like in PD.^18^, ^47^, ^57^

Within SKCM TCGA tissue biopsies, the difference in α‐syn and DJ‐1 gene expression between primary tumor and metastatic is not significant. This suggests that the involvement of these proteins in advanced metastatic melanoma may come through the protein level/PTMs^61^ and/or regulation of α‐syn aggregation and interaction with DJ‐1. Our hypothesis is further supported by the fact that there is only a significant positive correlation between alpha‐synuclein and DJ‐1 overexpression in primary and not in metastatic SKCM tumors and that α‐syn but not DJ‐1 elevation could deteriorate the clinical outcome of patients possibly by promoting metastasis, through an unknown so far mechanism.

To further characterize the protein expression in melanoma patients and investigate the metastatic potential, we explored the presence of the α‐syn aggregated forms in patients’ tissue samples. Since malignant melanoma can spread relatively quickly and metastasize through nearby lymph nodes,^1^, ^53^ we performed IHC expression analysis of α‐syn pathogenic forms and DJ‐1 in lymph nodes from metastatic melanoma and non‐metastatic controls (prostate neoplasm). Aggregated forms of α‐syn were detected in metastatic melanoma lymph nodes. This result supports the possible role of α‐syn aggregation in melanoma progression and metastasis and its potential as a biomarker for lymph node metastasis, although additional studies using quantitative approaches will be necessary in a larger cohort of patients to confirm this initial finding. A similar behavior was observed for other cancer‐related proteins; for instance, misfolding and prion‐like amyloid aggregation of p53 seem to play a crucial role in cancer development^62^ with the misfolded/aggregated states of mutant p53 representing prospective therapeutic targets. Although changes in α‐syn did not distinguish malignant and benign melanocytic skin lesions^61^ they may be useful for the diagnosis of metastatic melanoma, especially through the aggregated forms. Further studies in larger cohorts of metastatic melanoma patient samples will be required to assess the potential of α‐syn as a histological biomarker in the clinical setting.

Given the extensive work on α‐syn S129 phosphorylation as a pathological hallmark in PD,^63^ we investigated whether this PTM was also altered in melanoma. Interestingly, we observed almost no detection of α‐syn‐S129 phosphorylation in melanoma lymph nodes. Recent studies suggest that some PTMs seen in pathological aggregates (such as pS129) may occur after α‐syn aggregation or inhibit fibril formation^24^ as a consequence, rather than a cause for synucleinopathies. Thus, this may also be the case in melanoma. Since the effects of α‐syn‐S129 phosphorylation are still controversial,^21^, ^24^ and other phospho‐forms may also play a role in melanoma, further studies exploring the global α‐syn phosphorylation patterns and their role in melanoma progression are needed.

Our study showed that α‐syn is overexpressed in human melanoma tissues and melanoma cell lines, in agreement with previous studies.^61^, ^64^ α‐syn protein levels were higher in SK‐MEL‐28 compared to A375 cells and intracellular. In contrast, the A375 melanoma cell line expressed intracellular α‐syn at low levels, with parallel low secretion of the protein.

SK‐MEL28 cells can be traditionally considered more aggressive and metastatic than A375 cells. However, these cells have low invasive potential and recent in vitro studies suggest that A375 cells display a higher proliferation, migration, and invasion rate than SK‐MEL28 associated with higher matrix metalloproteinase‐2 (MMP2) enzymatic activity.^65^ In PD, it has been suggested that α‐syn pathology can spread from one cell type to another either through direct transfer or induction and, notably, from cancer cells such as glioblastoma cells to normal cells such as astrocytes.^66^ Induced or ‘received’ α‐syn is associated with an increase of oncogenic/stem cell markers in astrocytes, suggesting that the spreading of α‐syn in a ‘prion‐like’ manner may also take place in certain types of cancer, as proposed in synucleinopathies.^67^

From previous studies and our own results, we hypothesize that these two cell types (A375 and SK‐MEL‐28) can both have α‐syn‐dependent migrative or metastatic potential using distinct molecular mechanisms. DJ‐1, in contrast, is highly expressed intracellularly in both melanoma cell lines. These preliminary findings suggest that α‐syn and DJ‐1 may play a role in melanoma progression possibly by participating in common cell signaling pathways. For instance, the upregulation of DJ‐1, an already‐known oncogene^13^, ^15^ in melanoma, was shown to regulate PTEN/AKT pathway for cell survival and migration.^59^ Additionally, or alternatively, DJ‐1 may play a protective role in attenuating the α‐syn aggregation at a later stage like in PD.^14^

In silico PPIs and molecular docking revealed DJ‐1 as one of the most potent stable partners for α‐syn. Molecular docking is a computational method that can predict the binding mode and free energy of a ligand (protein or small molecule) to a protein.^68^ According to our results, there is indeed a strong interaction between α‐syn and DJ‐1 forming a stable complex (global binding energy −17.13 kcal/mol) with the domain including VAL‐52 for α‐syn to interact with DJ‐1 (through LEU‐75) proved to be in a highly hydrophobic α‐syn region (VAL‐48, VAL‐49, HIE‐50, ‐GLY‐51, VAL‐52).

N‐terminal residues in α‐syn (from 36 to 42 and 45 to 57 aa) are very critical for nucleation of aggregation,^29^ supporting the notion that DJ‐1 may interact with α‐syn via one of the aggregation domains, possibly offering a protective role for aggregation. The α‐syn‐associated overexpression of DJ‐1 observed in melanoma cells may enhance its binding to the α‐syn aggregation domain. This may constitute a protective mechanism in melanoma cells to protect α‐syn from aggregation, a mechanism that has been suggested in PD.^69^ The α‐syn and DJ‐1 association in melanoma cells further agrees with the positive correlation in melanoma patients indicating that their combined role could be a useful diagnostic and prognostic biomarker that needs to be further explored.

The high incidence and mortality rate of malignant melanoma could be partly due to the failure of current therapies.^70^ Of note, the DNA alkylating agents, dacarbazine, and its analog temozolomide that are used to treat metastatic melanoma,^4^, ^5^ demonstrate relatively low patient response. Characterizing the biological molecules and signaling pathways involved in chemotherapy sensitivity would be helpful for selecting therapeutic schemes and evaluating prognosis for melanoma. We, therefore, explored whether chemotherapeutic agents used in melanoma treatment, such as temozolomide, may affect the expression levels and/or PPIs between α‐syn and DJ‐1, thus these proteins may be implicated in common pathways involved in melanoma chemosensitivity. We have also explored more general potent chemotherapeutic drugs such as doxorubicin, which has already been found to co‐localize with α‐syn aggregates suggesting an interaction of doxorubicin with α‐syn in PD.^29^

Temozolomide was chosen as the most potent drug for SK‐MEL‐28 among other melanoma cell lines, according to the IC50 values (GDSC datasets). Both α‐syn and DJ‐1 protein levels reduced in the presence of temozolomide in SK‐MEL‐28 melanoma spheroids suggesting that α‐syn and/or DJ‐1 may be implicated in common mechanisms underlying melanoma chemosensitivity, the toxic effect of temozolomide or signaling pathways that reverse chemo‐resistance.^71^ This simultaneous reduction may be caused by: (i) the direct effect of the drug binding to the DNA thus affecting transcription, (ii) temozolomide binding to each protein and/or the complex (α‐syn/DJ‐1) or (iii) the stimulation of the degradation of both proteins by the lysosome or proteasome, thereby affecting protein degradation and stability. Future studies should investigate whether the modulation of the protein levels by the temozolomide takes place at the transcriptional or post‐transcriptional level, and test the physical interaction between the drug and the proteins. Temozolomide‐mediated regulation of α‐syn levels may also prevent α‐syn aggregation in melanoma and given that DJ‐1 is also decreased, an investigation of the interaction of both proteins in the presence of the drug should be performed.

Previous studies used molecular docking to predict the binding affinity of small molecule inhibitors to protein targets implicated in PD and other diseases.^72^, ^73^ To the best of our knowledge, the relationship, correlation, and interaction of α‐syn and DJ‐1 in melanoma have not been explored so far, which motivated us to explore the possibility of conducting molecular docking using chemotherapeutic drugs to target our proteins of interest alone or as a complex. Among the three drugs, doxorubicin formed the more stable interaction for both proteins and the complex through α‐syn, followed by temozolomide and dacarbazine.

Doxorubicin was found to induce the early onset of secondary structural changes from random coil to β‐sheet in the α‐syn leading to its aggregation^29^ and this may be the case for temozolomide that may cause eventually the degradation of the misfolded/aggregated protein, which needs to be further explored. As previously shown in other cellular models, α‐syn interacts with DJ‐1.^14^ Nevertheless, additional studies should verify the interaction between the two proteins in melanoma cells. In addition, in vitro studies will be necessary in order to confirm the proposed binding of TMZ to α‐syn for example using NMR, and to assess the effect on α‐syn fibrillization, as detected by thioflavin T assay.

In melanoma, increased DJ‐1 levels and interactions with α‐syn may modulate α‐syn aggregation, suggesting a novel potential therapeutic approach. Development of future drugs that selectively prevent the α‐syn‐DJ‐1 interactions may therefore represent an opportunity to re‐sensitize melanoma tumors to standard chemotherapeutic drugs.^71^

Overall, we posit that novel potential biomarkers such as α‐syn and/or DJ‐1 may help diagnose patients with early‐stage melanoma, who are likely to develop advanced metastatic disease and would benefit from additional therapies. Investigation of the α‐syn/DJ‐1 involvement in melanoma progression and chemosensitivity could prove beneficial for the discovery of novel therapeutic targets that will improve current treatments.

### 4.1 Message of manuscript

α‐syn and DJ‐1 are upregulated in primary and metastatic SKCM. Aggregated α‐syn was selectively detected in metastatic melanoma lymph nodes. α‐syn‐associated overexpression of DJ‐1 in melanoma cells is consistent with a positive correlation in melanoma patients, supporting PPI. Molecular docking identified a stable protein complex, with differential binding to chemotherapy drugs, opening novel perspectives for therapeutic intervention.

## AUTHOR CONTRIBUTIONS

**Agathe Quesnel:** Data curation (equal); formal analysis (equal); investigation (equal); methodology (equal); writing – original draft (equal); writing – review and editing (equal). **Leya Danielle Martin:** Data curation (equal); formal analysis (equal); methodology (equal); writing – review and editing (equal). **Chaimaa Tarzi:** Data curation (equal); formal analysis (equal); investigation (equal); methodology (equal); writing – original draft (equal); writing – review and editing (equal). **Vasileios P. Lenis:** Data curation (equal); formal analysis (equal); writing – review and editing (equal). **Nathan Coles:** Methodology (equal); writing – review and editing (equal). **Meez Islam:** Investigation (equal); supervision (equal); writing – review and editing (equal). **Claudio Angione:** Formal analysis (equal); investigation (equal); methodology (equal); supervision (equal); writing – review and editing (equal). **Tiago F. Outeiro:** Conceptualization (equal); investigation (equal); methodology (equal); writing – review and editing (equal). **Ahmad A. Khundakar:** Formal analysis (equal); investigation (equal); methodology (equal); supervision (equal); writing – review and editing (equal). **Panagiota S. Filippou:** Conceptualization (equal); data curation (equal); investigation (equal); methodology (equal); supervision (lead); writing – original draft (equal); writing – review and editing (equal).

## FUNDING INFORMATION

PSF is supported by seed corn funding from Teesside University and the Biochemical Society Eric Reid Fund for Methodology Grant. TFO is supported by the Deutsche Forschungsgemeinschaft (DFG, German Research Foundation) under Germany's Excellence Strategy—EXC 2067/1‐390729940.

## CONFLICT OF INTEREST STATEMENT

The authors declare no conflicts of interest.

## ETHICAL APPROVAL AND CONSENT TO PARTICIPATE

Sample collection was approved by the NovoPath Biobank Newcastle Research Ethics Committee (REC Ref 17/NE/0070). All procedures followed the Declaration of Helsinki. Patient‐informed consent was provided under the existing ethics approval procedure.

## CONSENT FOR PUBLICATION

All authors consent to the publication of this manuscript.

## Supporting information


Data S1.

## Data Availability

The datasets generated and/or analyzed during the current study are available from the corresponding author on reasonable request.
