# Supplementary material for: Uncovering potential diagnostic and pathophysiological roles of α‐synuclein and DJ‐1 in melanoma
Source: Cancer Med. 2024 Jan 8;13(1):e6900. doi: 10.1002/cam4.6900 (PMC10807602; doi:10.1002/cam4.6900)
Supplement: Supplementary file 1 — Data S1. [file CAM4-13-e6900-s001.docx]

**A**


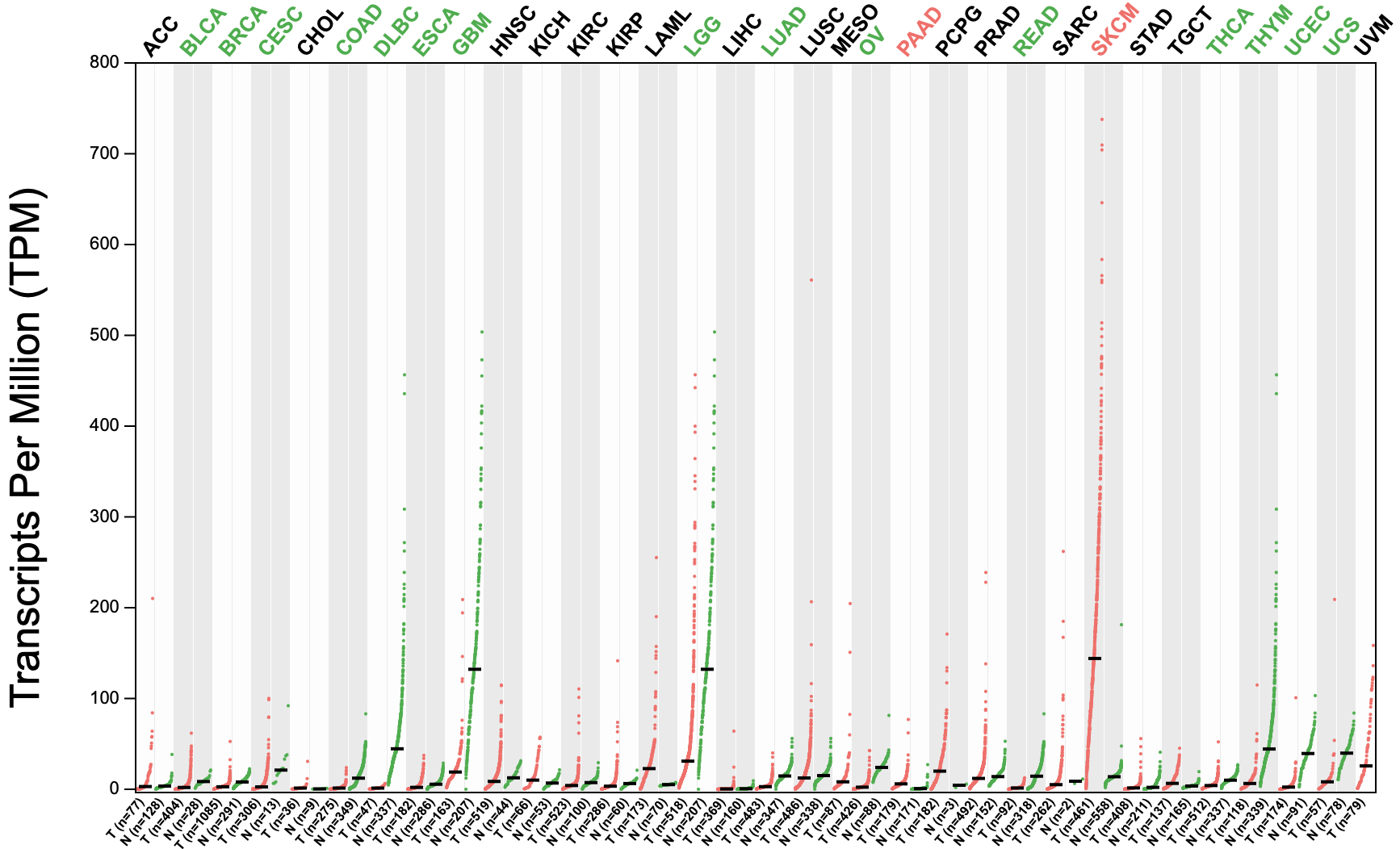


**B**


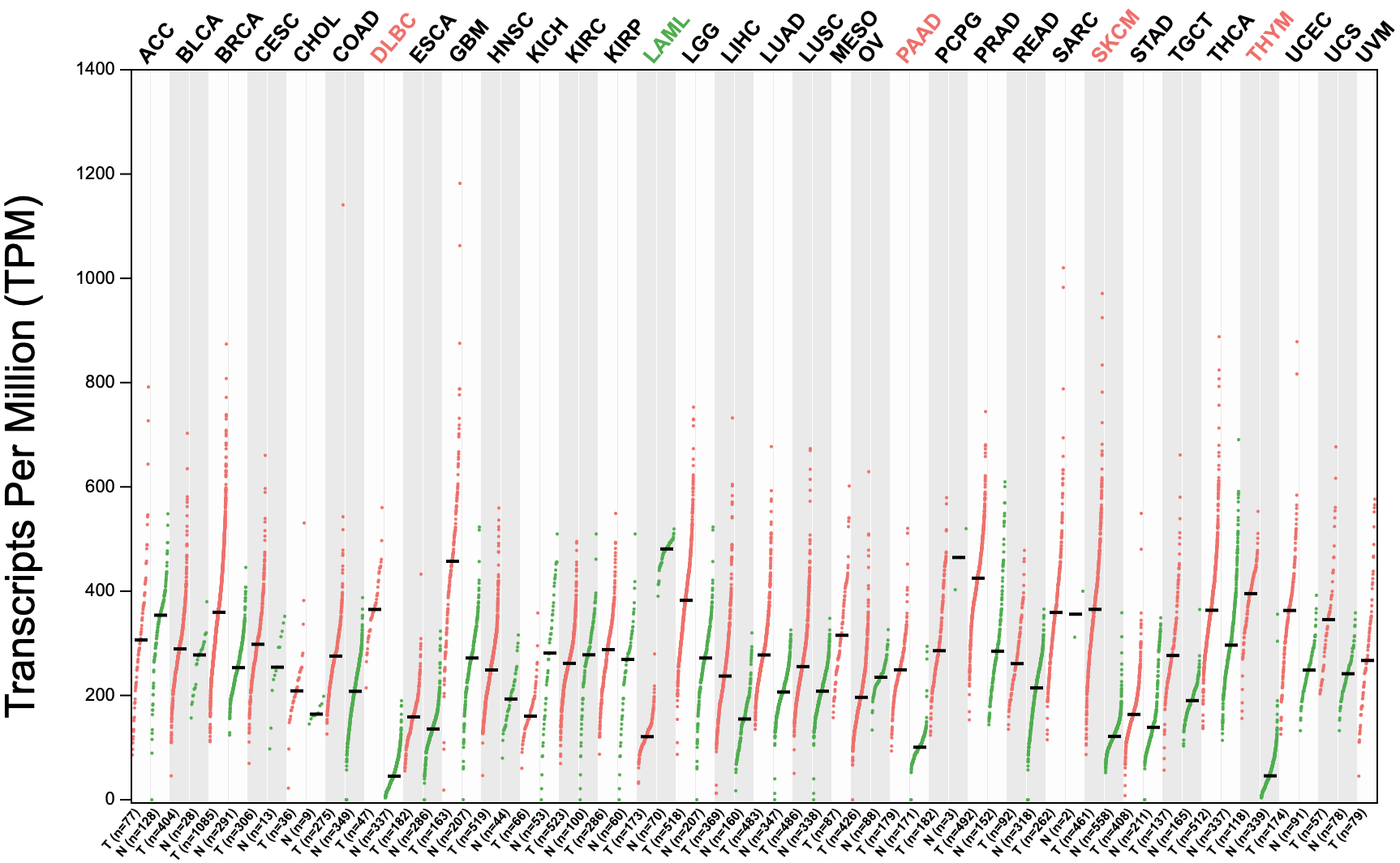


**Figure S1. Expression profiles of *SNCA* and *PARK7* in different cancer types.** *SNCA* (**A**) and *PARK7* (**B**) gene expression (transcripts per million; TPM) are indicated across tumour and normal samples by the TCGA/GTEx data source available within GEPIA2. **(A)** Compared to normal tissues, the expression of *SNCA* was significantly upregulated in skin cutaneous melanoma (SKCM) samples and the upregulation is very conspicuous compared with that in other kinds of cancer. **(B)** *PARK7*, had less selectivity for SKCM higher expression with a similar upregulation in DLBC, PAAD, SKCM and THYM. Each dot represents a distinct tumour or normal sample. Differential expression of each gene is indicated as upregulation (red) and downregulation (green). Values from RNAseq data represented as transcripts per million (TPM) indicating the gene expression profile across all tumor samples and paired normal tissues (Dot plot). Abbreviations: ACC: adrenocortical carcinoma; BLCA: bladder urothelial carcinoma; BRCA: breast invasive carcinoma; CESC: cervical squamous cell carcinoma; CHOL: cholangiocarcinoma; COAD: colon adenocarcinoma; DLBC: lymphoid neoplasm diffuse large B cell lymphoma; ESCA: esophageal carcinoma; GBM: glioblastoma multiforme; LGG: brain lower grade glioma; HNSC: head and neck squamous cell carcinoma; KICH: kidney chromophobe; KIRC: kidney renal clear cell carcinoma; KIRP: kidney renal papillary cell carcinoma; LAML: acute myeloid leukemia; LIHC: liver hepatocellular carcinoma; LUAD: lung adenocarcinoma; LUSC: lung squamous cell carcinoma; MESO: mesothelioma; OV: ovarian serous cystadenocarcinoma; PAAD: pancreatic adenocarcinoma; PCPG: pheochromocytoma and paraganglioma; PRAD: prostate adenocarcinoma; READ: rectum adenocarcinoma; SARC: sarcoma; SKCM: skin cutaneous melanoma; STAD: stomach adenocarcinoma; TGCT: testicular germ cell tumors; THCA: thyroid carcinoma; THYM: thymoma; UCEC: uterine corpus endometrial carcinoma; UCS: uterine carcinosarcoma; and UVM: uveal melanoma; LAML: acute myeloid leukemia; Lung_AC: lung adenocarcinoma; Lung_SC: lung squamous cell carcinoma; Renal_CC: renal clear cell carcinoma; Renal CH: renal chromophobe cell carcinoma; Renal PA: renal papillary cell carcinoma; Uterus_CS: uterine carcinosarcoma; Uterus_EC: uterine corpus endometrial carcinoma.


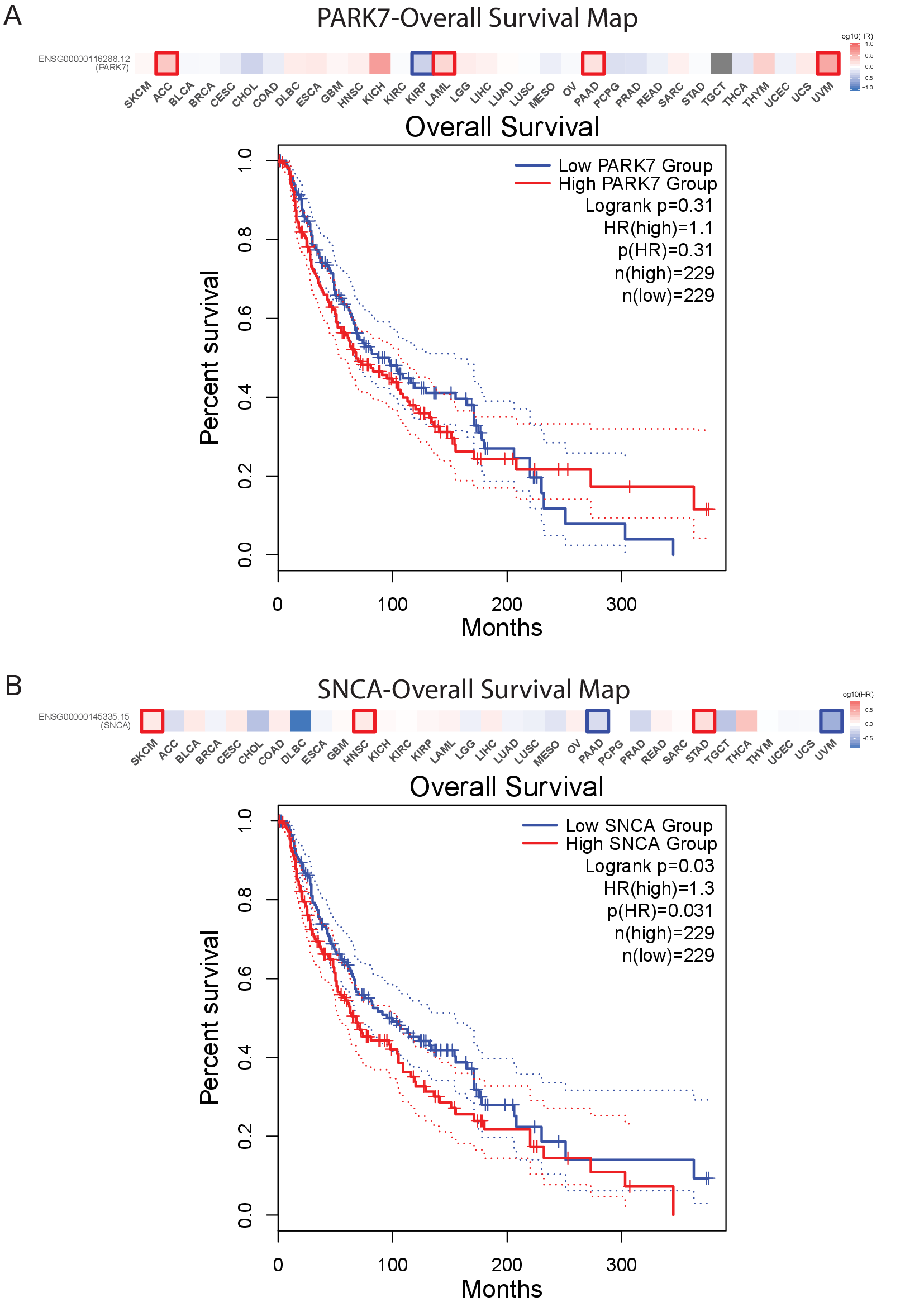


**Figure S2. Correlation between *SNCA* and *PARK7* gene expression with overall survival (OS) prognosis across different cancers including SKCM.** The correlation of *PARK7* **(A)** and *SNCA* **(B**) gene expression with OS for each cancer type is indicated in the cancer survival map (TCGA data, created with GEPIA2). Kaplan-Meier curves depicting the percent survival based on overall survival (OS) in months for SKCM patients are also indicated with high (red) and low (blue) expression levels of *PARK7* **(A)** and *SNCA* **(B).** In SKCM high alpha-synuclein (*SNCA*) expression was linked to poor prognosis (Logrank p=0.03), whereas no significant correlation was observed for *PARK7* (Logrank p=0.31).


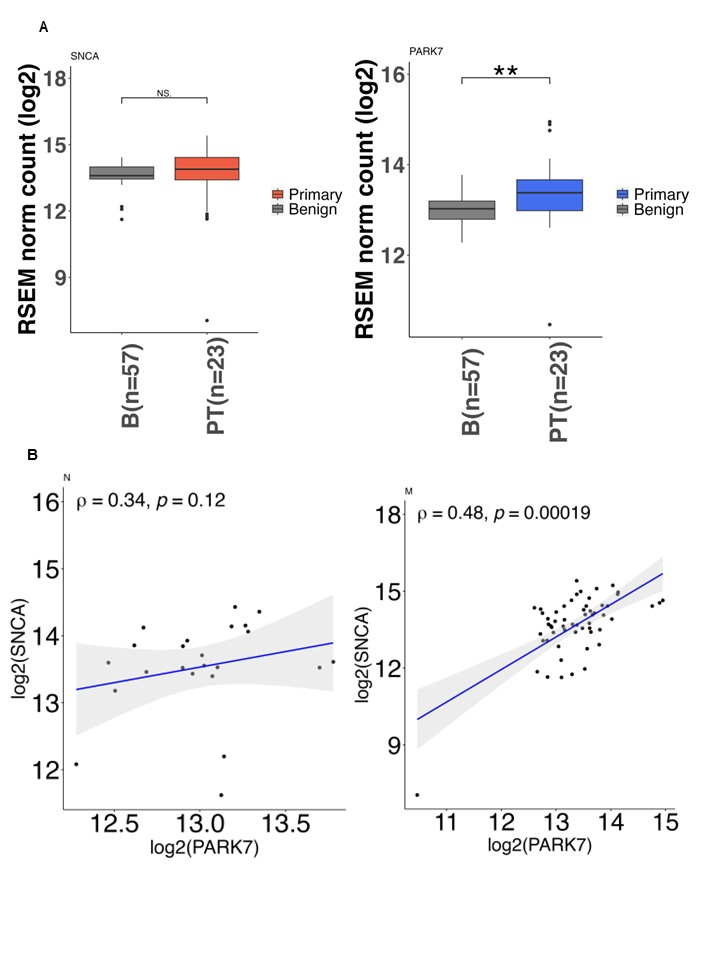


**Figure S3. Expression profiles of *SNCA* and *PARK7* in primary melanomas (n=57) and in benign** **melanocytic nevi (n=23). (A)** RNA sequencing data were obtained from GEO (GSE112509)[46] and analyzed using the Wilcoxon Rank Sum test with R software. *PARK7* was significantly upregulated in primary melanomas compared to benign, whereas not *SNCA*. **(B)** Spearman correlation analysis revealed a good positive correlation between *SNCA* and *PARK7 (* ρ = 0.48, p-value = 0.00019) in primary melanomas (right panel) but not in benign melanocytic nevi (ρ = 0.34, p-value = 0.12)(left panel).

**
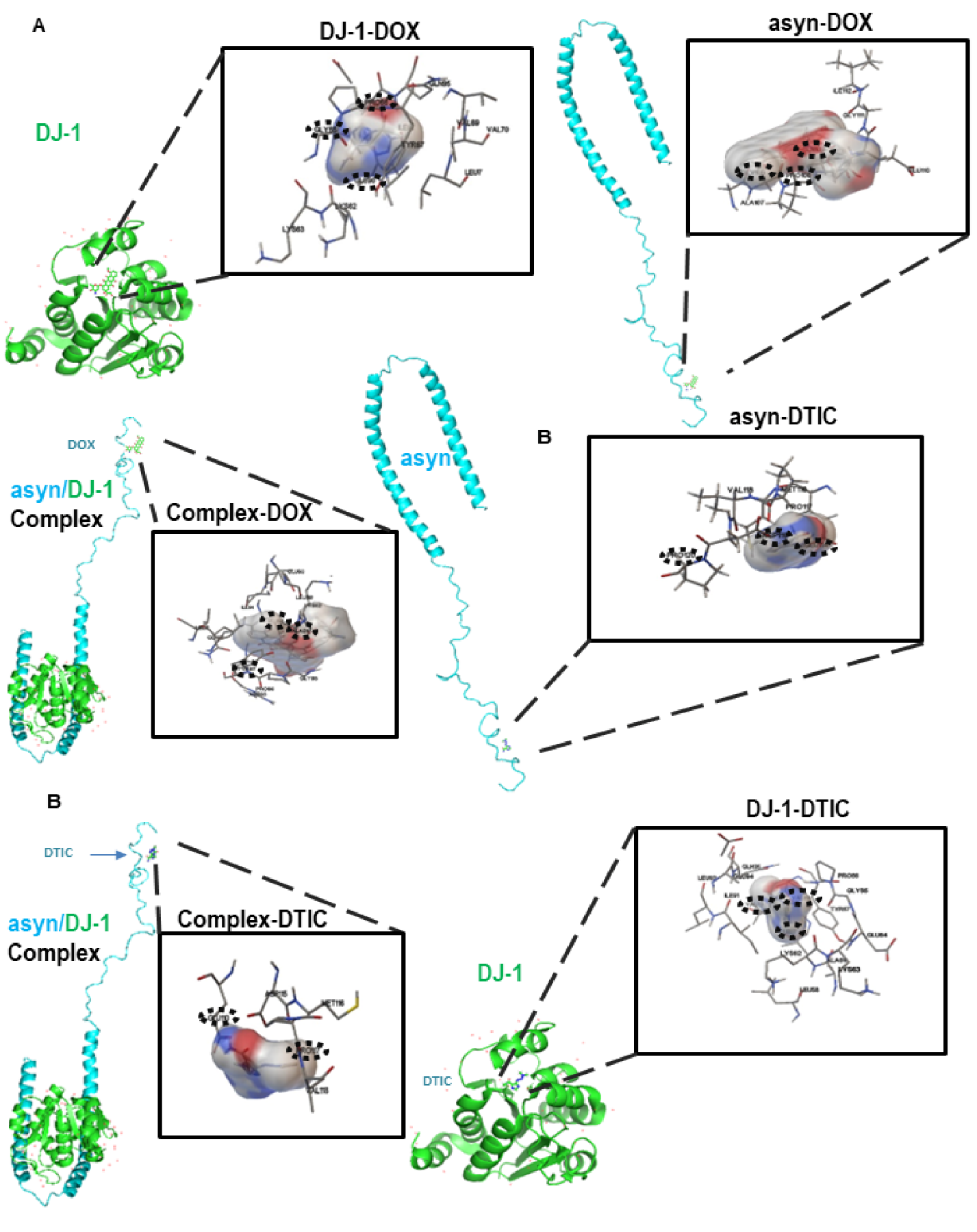
Figure S4. Molecular docking of the chemotherapeutic drugs** **dacarbazine and doxorubicin with alpha-synuclein and/or DJ-1.** Illustration (cartoon) of doxorubicin (DOX) (**A**) and dacarbazine (DTIC) (**B**) targeting α-syn, DJ-1 or their complex and their ligand-targeted and amino acids involved. The interacting amino acids (circled-indicated residues) for DTIC are: α-syn -DTIC (GLU 114, ASP 119, PRO 120), DJ-1-DTIC (VAL 69, VAL 70, LEU7) and complex - DTIC (GLU 110, PRO 117). The interacting amino acid residues for DOX are: α-syn-DOX (GLY 106, GLN 109, PRO 108), DJ-1-DOX (GLY 65, GLU 90, PRO 66) and complex-DOX (ALA 61, GLU 94, TYR 67).

**Table S1.** Ranking of the multi-member conformational clusters out of 10 molecular docking runs for protein-protein interaction of alpha-synuclein and DJ-1 complex formation. The following parameters were explored: the binding energy of the complex (global energy; glob), the softened attractive (aVdW) and repulsive(rVdW) van der Waals energy, the atomic contact energy (ACE), the insider measure (inside), the attractive (aElec) and repulsive (rElec) short-range Coulomb electrostatics, as well as the attractive (laElec) and repulsive (lrElec) long-range Coulomb electrostatics, the hydrogen and disulphide bonds (HB), the PI-PI (piS), the cation-PI stacking (catpiS) and the aliphatic interactions (aliph).

| **rank** | **glob**  **(kcal/mol** | **aVdW** | **aVdW** | **ACE** | **inside** | **aElec** | **rElec** | **laElec** | **lrElec** | **HB** | **piS** | **catpiS** | **aliph** |
| --- | --- | --- | --- | --- | --- | --- | --- | --- | --- | --- | --- | --- | --- |
| 2 | **-17.13** | -20.05 | 4.97 | 0.39 | 15.3 | -33.93 | 45.18 | -12.08 | 10 | -2.4 | 0 | 0 | -3.5 |
| 10 | -5.47 | -7.77 | 5.18 | 1.43 | 3.29 | 0 | 0 | 0 | 0 | 0 | -1.5 | -1.5 | 0 |
| 3 | 3.91 | -24.1 | 16.17 | 1.27 | 17.73 | -26.51 | 34.98 | -30.03 | 34.63 | -1.45 | 0 | 0 | -4.5 |
| 7 | 4.89 | -23.09 | 32.74 | 0.47 | 14.9 | -70.06 | 49.98 | -3.47 | 13.51 | -2.46 | 0 | 0 | 0 |
| 8 | 13.35 | -5.97 | 2.13 | 8.12 | 11.82 | -3.43 | 0 | -18.28 | 5.49 | 0 | 0 | 0 | -1.5 |
| 1 | 13.89 | -26.15 | 11.58 | 18.89 | 14.44 | -67.53 | 23.48 | -36.41 | 30.29 | -3.66 | 0 | 0 | -1.5 |
| 5 | 29.48 | -9.32 | 0.8 | 8.23 | 4.49 | -21.9 | 61.47 | -6.55 | 28.09 | -0.99 | 0 | 0 | 0 |
| 4 | 365.39 | -21.04 | 511.74 | -8.72 | 8.77 | 0 | 0 | 0 | 0 | -0.73 | 0 | 0 | -1.5 |
| 6 | 410.6 | -23.07 | 506.86 | 14.27 | 5.69 | -7.98 | 64.36 | 0 | 5.6 | -1.29 | 0 | 0 | -0.5 |
| 9 | 530.06 | -14.41 | 687.8 | 3.43 | 7.55 | 0 | 37.64 | 0 | 1.93 | -1.93 | 0 | 0 | -6.5 |

**Table S2. Parameters related to the distinct residues, where: "R-A" and "L-B" stand for ‘receptor’ (α-synuclein) and ‘ligand’ (DJ-1), respectively using the MM/GBSA method.** The following parameters are indicated: Van der Waals potential (VDW), electrostatic potential (ELE), Polar Solvation free energies predicted by the Generalized Born model (GB), Nonpolar contribution to the solvation free energy calculated by an empirical model (SA). ‘TOTAL’ corresponds to the final estimated binding free energy (with an energy less than -0.05) (kcal/mol) calculated from the above parameters. The higher the negative binding free energy, the higher the stability of the interacting residues.

| **RESIDUE_ID** | **VDW** | **ELE** | **GB** | **SA** | **TOTAL**  **(kcal/mol)** |
| --- | --- | --- | --- | --- | --- |
| **R-A-VAL-52** | -3.01 | -6.92 | 6.86 | -0.67 | -3.74 |
| **R-A-VAL-48** | -2.12 | -4.67 | 4.42 | -0.39 | -2.75 |
| **L-B-LEU-75** | -2.09 | -1.03 | 1.23 | -0.66 | -2.55 |
| **R-A-ALA-56** | -1.18 | -0.29 | -0.49 | -0.24 | -2.2 |
| **R-A-VAL-49** | -1.71 | -1.79 | 1.95 | -0.32 | -1.87 |
| **R-A-VAL-26** | -1.43 | 0.52 | -0.5 | -0.3 | -1.71 |
| **R-A-VAL-15** | -1.6 | 1.2 | -0.97 | -0.31 | -1.68 |
| **R-A-ALA-11** | -1.55 | -0.62 | 0.79 | -0.25 | -1.63 |
| **L-B-LEU-126** | -1.16 | -0.72 | 1.12 | -0.43 | -1.2 |
| **L-B-ALA-84** | -0.98 | 0.98 | -0.71 | -0.36 | -1.06 |
| **L-B-GLY-134** | -2.29 | -0.03 | 1.71 | -0.43 | -1.04 |
| **R-A-THR-22** | -1.99 | -3.45 | 4.99 | -0.52 | -0.97 |
| **R-A-LYS-60** | -0.68 | -35.48 | 35.54 | -0.23 | -0.85 |
| **R-A-ALA-53** | -0.61 | -2.13 | 2.04 | -0.08 | -0.79 |
| **R-A-ALA-18** | -0.74 | -1.44 | 1.69 | -0.25 | -0.74 |
| **R-A-VAL-74** | -0.61 | 0.5 | -0.37 | -0.21 | -0.7 |
| **L-B-PRO-41** | -1.17 | -1.25 | 2.04 | -0.27 | -0.64 |
| **L-B-ALA-37** | -0.98 | -2.03 | 2.72 | -0.31 | -0.59 |
| **L-B-HIE-113** | -0.41 | 0.02 | -0.2 | 0 | -0.59 |
| **L-B-GLY-135** | -0.55 | 0.31 | -0.21 | -0.08 | -0.53 |

**Table S3. Temozolomide (TMZ) pan-cancer drug sensitivity in a variety of cancer cell lines (GDSC project web portal).** (IC50 drug sensitivity data extracted from [https://www.cancerrxgene.org/](about:blank)).

| **Cell Line** | **TGCA Class** | **Tissue** | **Tissue Sub-Type** | **IC50** | **AUC** |
| --- | --- | --- | --- | --- | --- |
| OCI-LY7 | DLBC | Blood | B_cell_lymphoma | 4.303994 | 0.712218 |
| JVM-3 | CLL | blood | Lymphoid_neoplasm_other | 9.463197 | 0.785896 |
| WIL2-NS | UNCLASSIF | blood | Lymphoid_neoplasm_other | 9.869702 | 0.800938 |
| DEL | UNCLASSIF | blood | Lymphoid_neoplasm_other | 9.917336 | 0.814904 |
| JSC-1 | DLBC | blood | B_cell_lymphoma | 10.19236 | 0.803283 |
| **SK-MEL-28** | **SKMC** | **skin** | **melanoma** | **10.19508** | **0.761605** |
| SUP-M2 | UNCLASSIF | blood | anaplastic_large_cell_lymphoma | 10.55499 | 0.837194 |
| L-540 | UNCLASSIF | blood | Hodgkin_lymphoma | 11.78394 | 0.821505 |
| ATN-1 | ALL | blood | lymphoblastic_T_cell_leukaemia | 11.97232 | 0.832517 |
| QIMR-WIL | LAML | blood | acute_myeloid_leukaemia | 12.53711 | 0.84023 |
| HC-1 | UNCLASSIF | blood | hairy_cell_leukaemia | 13.67727 | 0.8286 |
| NKM-1 | LAML | blood | acute_myeloid_leukaemia | 14.21345 | 0.817737 |
| EoL-1-Cell | UNCLASSIF | blood | haematopoietic_neoplasm_other | 14.39638 | 0.862929 |
| SKM-1 | UNCLASSIF | blood | haematopoietic_neoplasm_other | 14.51031 | 0.850513 |
| G-MEL | SKCM | skin | melanoma | 14.58883 | 0.845335 |
| JVM-2 | CLL | blood | Lymphoid_neoplasm_other | 14.80063 | 0.809606 |
| SR | UNCLASSIF | blood | Lymphoid_neoplasm_other | 15.19008 | 0.864711 |
| IM-9 | MM | blood | myeloma | 16.15008 | 0.862187 |
| MOLM-16 | LAML | blood | acute_myeloid_leukaemia | 17.50694 | 0.864632 |
| SLVL | DLBC | blood | B_cell_lymphoma | 18.37007 | 0.844337 |
| SCC-3 | DLBC | blood | B_cell_lymphoma | 19.44336 | 0.885221 |
| CRO-AP2 | DLBC | blood | B_cell_lymphoma | 19.70855 | 0.883191 |
| KMS-12-BM | MM | blood | myeloma | 20.32784 | 0.889975 |
| MONO-MAC-6 | LAML | blood | acute_myeloid_leukaemia | 23.95759 | 0.881177 |

**Table S4. Temozolomide (TMZ) drug sensitivity with IC50 values in skin cutaneous melanoma (SKCM) cell lines.** Top 25 selected cell lines for TMZ chemosensitivity (IC50 drug sensitivity data extracted from [https://www.cancerrxgene.org/](about:blank)).

| **Cell Line** | **TGCA Class** | **Tissue** | **Tissue Sub-Type** | **IC50** | **AUC** |
| --- | --- | --- | --- | --- | --- |
| SK-MEL-28 | SKCM | skin | melanoma | 10.19508 | 0.761605 |
| G-MEL | SKCM | skin | melanoma | 14.58883 | 0.845335 |
| MZ7-Mel | SKCM | skin | melanoma | 50.50828 | 0.90587 |
| LOXIMVI | SKCM | skin | melanoma | 103.9737 | 0.942123 |
| COLO-783 | SKCM | skin | melanoma | 110.1312 | 0.936026 |
| Hs-940-T | SKCM | skin | melanoma | 111.7853 | 0.92115 |
| MEL-HO | SKCM | skin | melanoma | 141.8523 | 0.948918 |
| CP50-MEL-B | SKCM | skin | melanoma | 142.4287 | 0.957633 |
| LB2518-MEL | SKCM | skin | melanoma | 150.5044 | 0.952963 |
| RVH-421 | SKCM | skin | melanoma | 211.7879 | 0.960644 |
| VMRC-MELG | SKCM | skin | melanoma | 237.4869 | 0.943028 |
| RPMI-7951 | SKCM | skin | melanoma | 248.0642 | 0.974555 |
| M14 | SKCM | skin | melanoma | 269.7394 | 0.964616 |
| SK-MEL-1 | SKCM | skin | melanoma | 272.8272 | 0.95255 |
| Hs-939-T | SKCM | skin | melanoma | 287.4642 | 0.954101 |
| LB373-MEL-D | SKCM | skin | melanoma | 302.0132 | 0.974953 |
| SK-MEL-3 | SKCM | skin | melanoma | 303.5522 | 0.968113 |
| MEL-JUSO | SKCM | skin | melanoma | 319.5265 | 0.970589 |
| MZ2-MEL | SKCM | skin | melanoma | 342.0254 | 0.97233 |
| A101-D | SKCM | skin | melanoma | 386.4758 | 0.963794 |
| COLO-800 | SKCM | skin | melanoma | 395.7562 | 0.974537 |
| SK-MEL-31 | SKCM | skin | melanoma | 401.8347 | 0.958829 |
| IPC-298 | SKCM | skin | melanoma | 407.8559 | 0.975714 |
| GAK | SKCM | Skin | melanoma | 425.7099 | 0.979379 |

**Table S5.** Summary of the molecular docking runs for ligand(drug)-protein interaction and the parameters followed in the present study. The table displays the lowest energy docked conformation from each protein-drug interaction. For each ligand (Temozolomide (TMZ), Dacarbazine (DTIC), and Doxorubicin (DOX), the top-ranked Autodock conformational cluster is indicated, derived from the interaction between each ligand and the proteins alone (α-syn, DJ-1), or the complex.

| **Protein/Drug** | **Binding**  **Energy (kcal/mol)** | **Estimated**  **Inhibition Constant**  **(µM)** | **Ligand**  **Efficiency** | **Intermolecular**  **Energy (kcal/mol)** | **Electrostatic**  **Energy (kcal/mol)** | **Unbound**  **Energy (kcal/mol)** | **Reference**  **RMSD (Å)** | **Temp**  **(K)** |
| --- | --- | --- | --- | --- | --- | --- | --- | --- |
| **α-syn-TMZ** | -4.49 | 512.9 | -0.32 | -4.79 | -0.65 | 1.93 | 205.844 | 298.5 |
| **DJ-1-TMZ** | -4.87 | 271.34 | -0.35 | -5.16 | -0.13 | 1.84 | 120.29 | 298.5 |
| **Complex-TMZ** | -5.01 | 211.01 | -0.35 | -5.31 | -1.55 | 1.88 | 148.02 | 298.5 |
| **α-syn-DTIC** | -3.75 | 1780 | -0.29 | -5.24 | -1.83 | -0.23 | 205.16 | 298.5 |
| **DJ1-DTIC** | -3.75 | 1780 | -0.29 | -5.24 | -0.21 | -0.08 | 121.5 | 298.5 |
| **Complex-DTIC** | -3.81 | 1610 | -0.29 | -5.3 | -2.56 | -0.21 | 134.28 | 298.5 |
| **α-syn-DΟΧ** | -5.17 | 163.55 | -0.13 | -8.15 | -0.71 | 13.29 | 215.44 | 298.5 |
| **DJ-1-DΟΧ** | -6.19 | 29.06 | -0.16 | -9.17 | -024 | 13.77 | 115 | 298.5 |
| **Complex-DΟΧ** | -5.59 | 80.32 | -014 | -7.97 | -0.05 | 13.3 | 114.92 | 298.5 |
